# The role of myeloid cells in the pathogenesis of necrotizing enterocolitis; a scoping review

**DOI:** 10.3389/fped.2026.1750294

**Published:** 2026-02-27

**Authors:** Andrea Devaris, Alyssa M. Blaise, Liza Konnikova, Oluwabunmi Olaloye

**Affiliations:** 1 Department of Neonatology, Maternal Fetal Neonatal Institute, Johns Hopkins All Children’s Hospital, Saint Petersburg, FL, United States; 2 Department of Immunobiology, Yale School of Medicine, New Haven, CT, United States; 3 Human and Translational Immunology Program, Yale School of Medicine, New Haven, CT, United States; 4 Department of Obstetrics Gynecology and Reproductive Sciences, Yale School of Medicine, New Haven, CT, United States; 5 Department of Pediatrics, Yale School of Medicine, New Haven, CT, United States

**Keywords:** biomarkers, human milk, immune dysregulation, macrophages, monocytes, myeloid-derived suppressor cells, necrotizing enterocolitis, neutrophils

## Abstract

**Introduction:**

Necrotizing enterocolitis (NEC) is a severe gastrointestinal disorder that primarily affects preterm infants, resulting in significant morbidity and mortality. The exact cause of NEC remains unclear, but it is believed to involve a combination of immune dysregulation, intestinal injury, and microbiota imbalance.

**Methods:**

This scoping review examines existing human and animal studies that explore the role of myeloid cells (neutrophils, monocytes, macrophages, and myeloid-derived suppressor cells (MDSCs) in NEC pathogenesis.

**Results:**

A reduction in peripheral blood monocytes, along with increased infiltration of proinflammatory monocytes and neutrophils into the intestine, are strongly associated with NEC severity. Immunoregulatory MDSCs may provide protective benefits; however, their activity appears impaired in preterm infants with NEC. Therapies targeting these immune pathways, including transforming growth factor-β2 (TGF-β2) and lactoferrin, show promise in preclinical models for mitigating inflammation and improving outcomes in infants with NEC.

**Conclusions:**

Targeting myeloid cell immune responses represents a potential therapeutic strategy in NEC. Future research should focus on translating immune-modulating therapies to clinical practice, as such interventions may reduce NEC incidence and severity and offer new hope for vulnerable neonates.

## 1 Introduction

Necrotizing enterocolitis (NEC) is the most common devastating gastrointestinal emergency that causes significant morbidity and mortality rates in preterm infants (1, 2). Classical NEC occurs in 7%–12% of preterm infants most of whom are born before 32 weeks of gestation or with very low birth weight (VLBW, birthweight less than 1,500 g) (3, 4). Meanwhile, NEC can occur in late preterm or term infants with other comorbidities, including those with congenital cardiac defects (5). The precise cause of NEC remains elusive, although several risk factors have been identified, including prematurity, immune dysregulation, formula feeding, abnormal bacterial colonization of the gut, and intestinal ischemia (4). NEC symptoms can be insidious at first, but can quickly progress with hallmark signs on radiographic images such as pneumatosis intestinalis (air within the bowel wall), portal venous gas, and free air in the abdomen (6). The severity is characterized using modified Bell's staging system to classify infants into 1 of 3 stages of NEC severity based on systemic, radiographic and gastrointestinal findings (7). While some patients with NEC can be treated conservatively with antibiotics and withholding feeds, thirty to fifty percent of infants with NEC must undergo surgical intervention to remove compromised intestine (1). NEC is associated with multiple immediate and long-term complications, such as death, sepsis, intestinal failure, growth delay, and adverse neurodevelopmental outcomes (4) despite advances in neonatal care. Thus, highlighting the need for continued research to better understand its pathophysiology, improve diagnostic capabilities, and develop targeted therapeutic strategies.

Premature infants are particularly susceptible to ischemic insults, leading to mucosal damage, barrier dysfunction, and bacterial translocation, initiating the inflammatory cascade characteristic of NEC (8). Furthermore, proinflammatory mediators and chemo attractants trigger an infiltration of macrophages, neutrophils and myeloid-derived suppressor cells (MDSCs) that migrate from the blood into intestinal mucosa resulting in mucosal injury, barrier dysfunction, and systemic inflammation (9). MDSCs are immature myeloid cells with potent immunosuppressive functions that serve as a key component of neonatal immune defense and are essential for preventing excessive intestinal inflammation, including NEC (10). Reduced MDSC frequency and suppressive activity in preterm and very low birth weight infants correlate with increased NEC susceptibility and disease severity, highlighting impaired MDSC-mediated immune regulation as a contributor to NEC pathogenesis (10).

In healthy intestinal mucosa, macrophages are one of the first responders in infection, which are crucial for cytokine production, antigen presentation to other immune cells, and maintain homeostasis through the phagocytosis of debris and pathogens (11). Macrophages exist along a functional spectrum, broadly categorized as pro-inflammatory M1 cells that activate Toll-like receptor-4 (TLR4) signaling and drive intestinal injury, and anti-inflammatory M2 cells that promote immune regulation, epithelial repair, and resolution of inflammation (11). Monocytes circulate within the peripheral blood, while resident macrophages are localized to tissues including the intestine (11). Intestinal monocytes are broadly categorized into classical and non-classical subsets based on their surface marker expression and functional profiles. Classical monocytes, which express high levels of CD14 and low or no CD16 (CD14++CD16−), are primarily involved in phagocytosis and the initiation of inflammatory responses (12, 13). In contrast, non-classical monocytes, defined by lower CD14 and high CD16 expression (CD14 + CD16++), migrate along the endothelium and are typically involved in tissue surveillance and inflammation resolution under normal conditions (12, 13).

Neutrophils are a type of white blood cell that play a key role in the body's innate immune response, acting as the first line of defense against infection by rapidly migrating to sites of tissue injury or microbial invasion. In healthy individuals, they circulate in the blood and help maintain immune surveillance by identifying and neutralizing pathogens through phagocytosis, cytokine secretion, and the release of inflammatory mediators. They can also function as antigen-presenting cells to modulate downstream adaptive immune responses. In the context of NEC, a decline in circulating neutrophil count is often observed and is a critical indicator of disease severity and predictor of rapid progression (1, 8). Emerging evidence supports a role for neutrophils in maintaining intestinal immune homeostasis, suggesting that disruption of these protective functions may further contribute to intestinal injury and inflammation in NEC (8). Understanding the intricate roles of neutrophils, monocytes, macrophages, and MDSCs in both health and disease states is essential for unraveling the mechanisms underlying NEC. In this review, we delve into the multifaceted roles of these myeloid cells in NEC pathogenesis and intestinal homeostasis, while aiming to shed light on potential targeted preventive strategies and therapeutic interventions to mitigate the impact of this condition on premature infants.

## 2 Materials and methods

**Figure 1:**
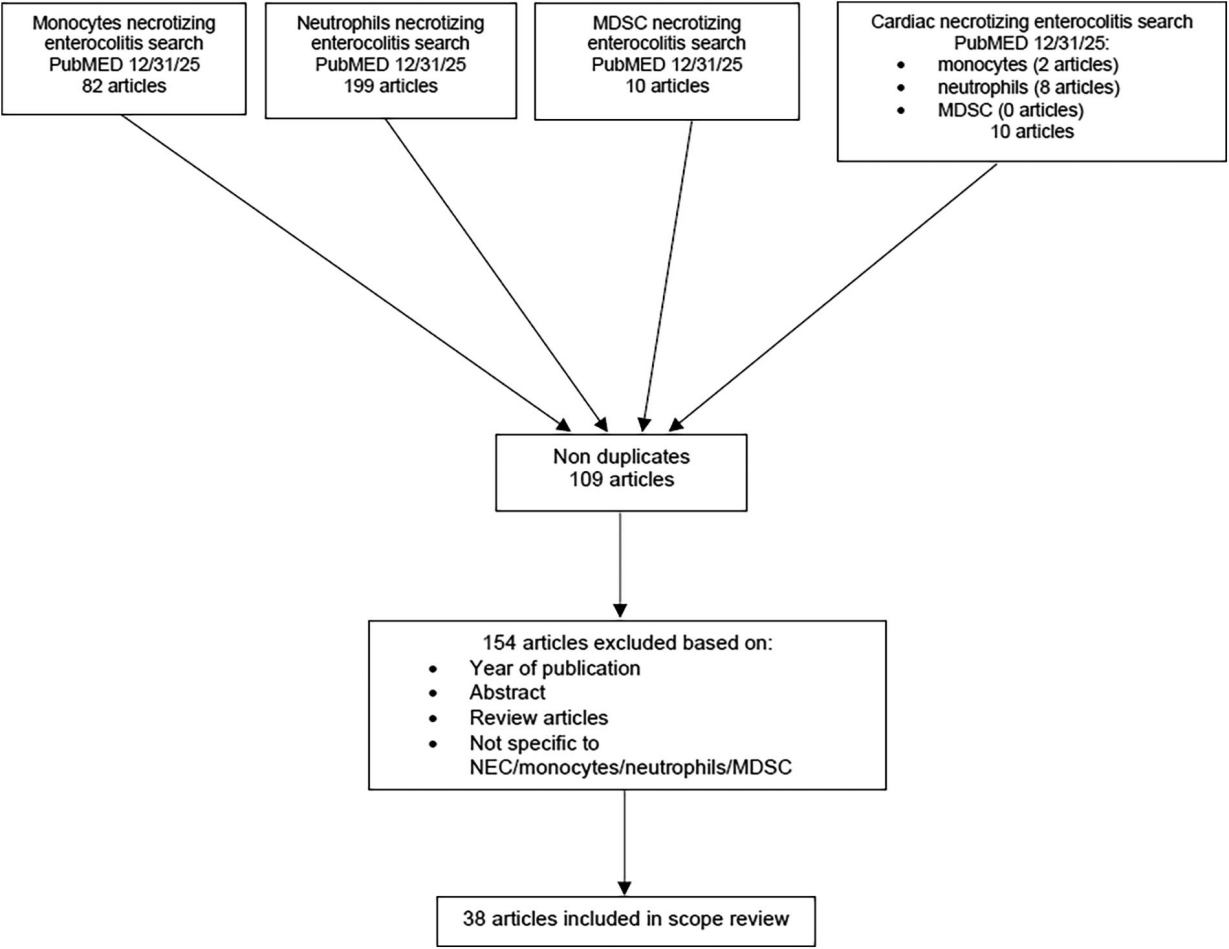
Flowchart of systematic review results.

For this scoping review of literature on the role of monocytes and neutrophils in the pathogenesis of NEC, we performed a search using PubMED with a timeframe spanning January 2011 through December 31, 2025 (60). The search was performed in separate attempts to capture unique research articles. We used the following strings in separate searches: “monocytes necrotizing enterocolitis” (82 articles), “neutrophils necrotizing enterocolitis” (199 articles), and “MDSC necrotizing enterocolitis” (10 articles). To highlight relevant studies capturing patients with congenital heart disease (CHD) who develop NEC we searched: “monocytes cardiac necrotizing enterocolitis” (2 articles), “neutrophils cardiac necrotizing enterocolitis” (8 articles), and “MDSC cardiac necrotizing enterocolitis” (0 articles), yielding a total of 109 unique articles. After all authors reviewed the abstracts, we excluded 154 articles that lacked relevance to the question, were outside the publication year range, or were review articles (Figure 1). Upon review of all articles, primary research findings that elucidate the role of neutrophils, monocytes, and MDSCs in the pathogenesis of NEC from 2011 to 2025 were summarized. We included 38 articles in total.

### 2.1 Relative decrease in peripheral absolute monocyte counts (AMC) as a clinical biomarker for classical NEC

NEC is characterized by a substantial infiltration of macrophages in the intestines, originating from monocytes in the blood, depleting peripheral blood monocytes to replenish the intestinal pool and respond to local inflammation (6). Several studies have investigated how changes in peripheral blood monocyte counts are altered in both medical (M-NEC) and surgical NEC (S-NEC) as complete blood counts are often obtained when the clinical suspicion for NEC is high (8, 14, 15). Absolute monocyte counts (AMC) are reduced in infants with NEC at the time of diagnosis compared to healthy infants and can be an adjunct biomarker to confirm the diagnosis of NEC (14, 16, 17). Wang et al. investigated AMC changes over time in infants with NEC, measuring values at birth, baseline, onset, and recovery (14). Their results showed a pronounced reduction at NEC onset, with median AMC in NEC infants significantly lower than controls [1.02 × 10^−9^/L [IQR 0.58–1.43] vs. 1.49 × 10^−9^/L [IQR 1.20–2.13], *p* < 0.001] (14). Importantly, no significant differences were seen at birth or baseline in infants with NEC when compared to healthy controls. Wang et al. proposed that this decline reflects migration of monocytes from the bloodstream to the intestine, where they differentiate into macrophages, consistent with the macrophage-rich histology of NEC (14).

When stratified by disease severity, AMC was significantly lower in infants with S-NEC than in those managed medically (M-NEC) [0.7 × 10^−9^/L [IQR 0.4–1.26] vs. 1.1 × 10^−9^/L [IQR 0.69–1.44], *p* = 0.005] (12). The percentage reduction was also more pronounced in S-NEC [−53.18% (IQR −74.98 to −17.70), *p* = 0.005] (14). This suggests that a profound decline in AMC at NEC onset, particularly reductions greater than 50%, may serve as a useful predictor of S-NEC and warrants closer clinical monitoring.

Infants with NEC had significantly lower AMC levels that were negatively correlated to NEC severity (Bell stage III vs. stage II) when compared to infants without NEC (9). Desiraju et al. further reinforced these findings by demonstrating a significant AMC drop in infants with advanced NEC (stage 3), establishing a 75% decrease from baseline as a threshold for stage 3 NEC, with 73% sensitivity and 87% specificity (18). This study confirmed that greater AMC reductions are associated with increased NEC severity, supporting AMC's role as a marker for disease progression and severity (18). Although there is currently no specific normal reference range for AMC in newborns of different gestational ages, a profound decrease in AMC of 50%–75% compared to baseline in preterm infants can be a useful clinical biomarker in NEC (14). Moroze et al. and Bisht et al. further demonstrated that tracking changes in AMC from a patient's baseline at the onset of illness enhances early recognition of NEC (16, 17). Monitoring declining AMC offers a practical, real-time adjunct biomarker to heighten clinical suspicion and support timely decision-making in infants at risk for severe NEC.

### 2.2 Transforming growth factor beta (TGF-β) and IL2 in peripheral blood of infants with classical NEC

Transforming growth factor-beta (TGF-β) and interleukin-2 (IL-2) are key cytokines involved in regulating intestinal homeostasis (19). TGF-β serves as a monocyte chemoattractant, activator and triggers differentiation into macrophages (19), promotes regulatory T cell (Treg) development, and limits pro-inflammatory T cell responses, while IL-2 is essential for T cell survival and the maintenance of Tregs, both of which help prevent excessive intestinal inflammation (20). Low levels of blood TGF-β and IL-2, along with high levels of IL-8, have been linked to an increased risk of NEC (20). Specifically, Maheshwari et al. found that TGF-β1 concentrations below 1,380 pg/mL predicted NEC with 64% accuracy, making it the first biomarker to estimate NEC risk in preterm infants (20). Their findings support earlier evidence that preterm neonates are more vulnerable to NEC due to a developmental deficiency of TGF-β isoforms (TGF-β1 and TGF-β2) in the intestine, which might lead to excessive macrophage-driven inflammation upon bacterial exposure (20). In parallel, diminished IL-2 signaling in NEC may further disrupt macrophage–T cell regulatory circuits, limiting Treg-mediated suppression of intestinal immune activation (20). Together, these data suggest that concurrent deficiencies in IL-2 and TGF-β signaling impair immune tolerance, promoting macrophage-driven intestinal inflammation in NEC.

TGF-β production by intestinal macrophages is higher in preterm infants compared to full-term infants with NEC (20). In S-NEC TGF-β was found to be linked with decreased tissue expression of TGF-β2 and decreased TGF-β bioactivity (20). This was recapitulated in neonatal mice, where disrupted TGF-β signaling exacerbated NEC-like mucosal injury, while enteral supplementation with recombinant TGF-β2 protected against such damage. This highlights TGF-β2's protective role against inflammatory mucosal injury in preterm intestines (20).

### 2.3 The pathogenic role of intestinal myeloid cells in human and experimental NEC

NEC is a heterogenous disease, and intestinal tissue samples are only available in infants who require surgery. Yet, several studies have highlighted the role of non-classical monocytes (CD14low/CD16high) and related cytokines in intestinal tissue and blood of infants with S-NEC (12, 13, 21). In infants with S-NEC, pro-inflammatory CD16 + CD163+ monocytes/macrophages were present in both peripheral blood and adjacent to blood vessels within the inflamed intestine suggesting migration from the peripheral blood to intestine (13). Additionally, interactions between CD16 + CD163+ monocytes/macrophages and T regs were prominent in S-NEC compared to neonates who required surgery for congenital intestinal anomalies (13). This implies that T regs attempt to suppress these monocytes/macrophages, or conversely, that the inflammation produced by CD16 + CD163+ monocytes/macrophages inhibits T reg cell function (13). Likewise, the presence of these monocytes within the intestine could explain the profound decrease in AMC that is well documented in S-NEC (13). Thus, significant accumulation of inflammatory non-classical monocytes in the intestinal tissues of infants with S-NEC results in impaired regulatory function and amplification of local inflammation and tissue damage (12).

Furthermore, in S-NEC, Colony Stimulating Factor 1 receptor + (CSF1R+) macrophages in NEC may overreact to bacterial stimuli resulting in epithelial damage (22). This inflammatory environment is further amplified by pro-coagulative environments. Balamurugan et al. demonstrated that platelet activation and subsequent integrin β3-mediated platelet-monocyte aggregation are key pathologic contributors to NEC (23). In murine models, the platelet specific knockout of integrin B3 led to inhibition of platelet-monocyte aggregates in circulating blood and intestines, thus reducing levels of inflammatory cytokines like TNF-α and IL1-β (23). These findings suggest that the interaction between platelets and monocytes is a key driver of intestinal injury in NEC and a potential therapeutic target.

The protein TLR4, a transmembrane receptor that detects pathogen-associated molecular patterns (PAMPs) including lipopolysaccharides (LPS) on bacteria has been shown to be impaired in S-NEC and animal models (24). Intestinal monocytes from infants with S-NEC exhibit higher TLR4 transcription compared to controls, and macrophages derived from these monocytes show increased pro-inflammatory cytokine production [Tumor Necrosis Factor alpha (TNF-α), interleukin-6 (IL-6)] with a reduction in anti-inflammatory cytokines (IL-10, TGF-β) (21). This imbalance may favor a pro-inflammatory environment and hinder Treg differentiation, as shown by reduced FOXP3 transcription in CD4+ T cells (21) which are known to drive intestinal inflammation in NEC (25).

However, not all monocyte subsets are involved in promoting intestinal injury; some play a regulatory role. A unique population of Neurolipin-1 (Nrp-1) high monocytes was identified in neonates and found to display potent immunosuppressive activity (26). Nrp1 binds to Sema4a activating the intracellular p38-MAPK/mTOR signaling axis to induce Nitric Oxide Synthase 2 (NOS2) transcription (26). This results in the production of nitric oxide, a key mediator of immunosuppression in monocytes. Notably, Nrp1 deficient myeloid cells resulted in worsening NEC severity, while transfer of Nrp1 high monocytes led to remission of NEC, identifying a specific immunosuppressive axis in neonatal monocytes that may provide a potential therapeutic strategy (26).

### 2.4 Myeloid-derived suppressor cells in the prevention of classical NEC

MDSCs are immature myeloid cells that are classified into two major subtypes: granulocytic/polymorphonuclear (PMN-MDSCs) and monocytic (M-MDSCs) (27). A primary feature of MDSCs is their potent ability to inhibit immune responses of T cells, B cells, and NK cells. Healthy, normal-weight infants (>2,500 g) had higher frequencies of MDSCs, and stronger suppressive activity compared to very low birth weight infants (<1,500 g) (2, 28). PMN-MDSCs in normal-weight infants displayed higher expression of nitric oxide (NO), prostaglandin E2 (PGE2), lactoferrin, and S100A8 and S100A9 proteins, which are calcium-binding proteins that form the heterodimer calprotectin which amplifies inflammation and is a biomarker of intestinal injury (28, 29). Low PMN-MDSC levels during the first three days of life were associated with a higher risk of developing NEC (2). Moreover, breastfed infants displayed higher levels of PMN-MDSCs compared to formula-fed infants, underscoring the role of breast milk in NEC prevention (2, 28).

Lactoferrin (LF), found in breast milk, plays a critical role in converting neutrophils and monocytes into potent MDSCs (LF-MDSCs) via NOS2, prostaglandin E2 (PGE_2_) and S100A8-A9 proteins (2, 28). These LF-MDSCs demonstrate superior anti-inflammatory and antibacterial activity compared to lactoferrin alone, and this effect has been observed in both preterm and full-term neonates (2). Importantly, breast milk–derived MDSCs (BM-MDSCs) can downregulate TLR4 on monocytes, a pathway that has been implicated in NEC pathogenesis (30). On the contrary, reduced MDSC activity in preterm or formula-fed infants can limit S100A8-A9 signaling, decreasing “stress tolerance” in monocytes and leaving the intestine vulnerable to inflammation (29). Thus, breast milk supports NEC protection by promoting higher levels of MDSCs and by enhancing calprotectin-mediated immune regulation (29, 30).

*In vivo* LF administration in newborn mice enhanced MDSC suppressive activity and immune regulation with evidence of substantially reduced bacterial load in the intestine and blood, suppressed inflammation, and prolonged survival with LF-MDSCs (2, 28). In newborn mice, adoptive transfer of these LF-induced MDSCs decreased bacterial burden, reduced inflammation, improved intestinal permeability, and increased survival during experimental NEC (28). Conversely, T cell transfer exacerbated inflammation and decreased survival, effects that were reversed by MDSC administration revealing MDSCs' inhibitory effect on T cells, in the context of experimental NEC (28). LF has a pivotal role in driving MDSC-mediated immune tolerance, highlighting LF-MDSCs as a promising therapeutic and preventive strategy for NEC.

Recently, NF-κB signaling has been identified as a central regulator of MDSC trafficking in NEC (10, 31). G-protein–coupled receptor kinase–interacting protein 2 (GIT2) is significantly upregulated in human and experimental NEC, with expression levels correlating positively with intestinal injury severity (10). Genetic deletion of *Git2* protects against NEC by enhancing intestinal recruitment of MDSCs through NF-κB–dependent upregulation of epithelial CXCL1 and CXCL12 (10). Depletion of MDSCs largely abolishes this protective effect, while increased MDSC accumulation restores adaptive immune balance by expanding regulatory T cells and suppressing Th17 responses (10).

Likewise, intestinal trefoil factor 3 (TFF3), a goblet cell-derived peptide involved in mucosal repair, promotes PMN-MDSC expansion and suppressive activity via NF-κB/COX2/PGE₂ signaling, with associated upregulation of S100A9, resulting in reduced intestinal inflammation, improved barrier permeability, decreased bacterial translocation, and improved survival in murine NEC (31). Finally, adenosine, present at higher concentrations in neonates than adults, protects against NEC by enhancing MDSC immunosuppressive and antibacterial functions and promoting intestinal trafficking through CXCR2-dependent mechanisms (32). Depletion of MDSCs or inhibition of their migration eliminates the protective effects of adenosine, underscoring the essential role of MDSCs in maintaining neonatal intestinal immune homeostasis (32).

### 2.5 Peripheral blood neutrophils as a predictive marker of severity in infants with NEC

Both clinical and experimental studies have explored the role of aberrant neutrophil responses in the progression and severity of NEC, highlighting neutrophils as both biomarkers of disease severity and active contributors to intestinal injury (1, 8, 33–36). Jiale Chen et al. developed a predictive model for the rapid progression of NEC in preterm neonates, identifying male sex, portal venous gas, neutropenia (<2.0 × 10^9^/L), and pH value <7.2 as risk factors driving the rapid progression of disease (surgical intervention or death within 48 h) (1). The model that included the four risk factors demonstrated an area under the curve (AUC) of 0.801, with 83% specificity and 66% sensitivity for rapidly progressive NEC (RP-NEC) which was associated with significantly higher mortality compared to nRP-NEC (1).

Furthermore, Pantalone et al. also noted sharp declines in absolute neutrophil counts (ANC) in infants ≥33 weeks at NEC onset, with neutrophil recovery following antibiotic treatment observed in surgical cases (8). Qin et al. reported a decreased in ANC at onset of NEC in neonates stating that the ANC was found to be the most sensitive predictor of severe surgical NEC, with a sensitivity of 71.7% (33). The decline in ANC was attributed to the migration of neutrophils from circulation to sites of inflammation, such as the bowel or peritoneum, highlighting their role in mediating the inflammatory response in NEC (1, 13). In contrast, Guo et al. demonstrated that infants who progressed to surgical intervention or death exhibited significantly higher ANC within 24 h of disease onset compared with infants managed medically (34). Predictive modeling identified ANC as a strong predictor of surgical or fatal NEC, and a composite model incorporating ANC, platelet-to-lymphocyte ratio (PLR), C-reactive protein (CRP), and procalcitonin (PCT) demonstrated good discriminatory performance (AUC 0.79) (34). Together, these findings suggest that dynamic changes in ANC may reflect both neutrophil tissue recruitment and escalating systemic inflammation, underscoring neutrophils as key contributors to NEC severity rather than passive inflammatory markers.

Mu et al. examined the neutrophil-to-lymphocyte ratio (NLR) in peripheral blood as a marker for NEC in preterm neonates (35). The study found that NLR was positively correlated with NEC severity and identified specific NLR values (≥3.20 and <1.6) in CBC 1 week prior to the diagnosis as predictive cutoffs for NEC risk (35). Higher NLR values and neutropenia were associated with NEC reflecting the inflammatory status in affected infants (35). Furthermore, elevated neutrophil and total leukocyte count have been shown to be independently correlated with systemic acidosis. In M-NEC and S-NEC peripheral neutrophil count had a significant negative correlation with systemic acidosis and intestinal ischemia, which are strongly associated with increased mortality (36). This supports the idea that neutrophils play a central role in NEC pathogenesis, both as a marker of disease severity and as contributors to the inflammatory environment. NLR may serve as a diagnostic tool for preterm NEC and can be employed at symptom onset to determine the inflammatory status in these infants (35). Together, these findings suggest that neutrophil-driven inflammation contributes to metabolic derangements and adverse outcomes, particularly in infants requiring surgical intervention.

Advances in high-resolution leukocyte phenotyping further highlight qualitative neutrophil changes as powerful diagnostic tools (37). Ferraro et al. demonstrated that neutrophil fluorescence intensity (NE-SFL), a marker of neutrophil immaturity and activation derived from automated cell population data, was significantly elevated at the onset of sepsis/NEC and outperformed traditional markers, including ANC, band counts, immature-to-total neutrophil ratio, and C-reactive protein (37). Elevated NE-SFL likely reflects mobilization of immature or highly activated neutrophils into the circulation, reinforcing the concept that neutrophil functional state may better capture NEC-associated inflammation than cell counts alone (37).

Collectively, these studies highlight the importance of peripheral blood neutrophil indices as predictors of NEC severity and clinical outcomes. Beyond their utility as biomarkers, neutrophils actively contribute to NEC pathophysiology by amplifying epithelial injury, ischemia, and systemic inflammation. Early recognition of neutrophil-driven inflammatory patterns may therefore support risk stratification, inform clinical decision-making, and help identify infants at increased risk for disease progression and the need for surgical intervention.

### 2.6 Neutrophils and neutrophil extracellular traps in driving intestinal inflammation

Given the drastic drops in peripheral neutrophil count associated with surgical NEC, several studies have explored pathways driving tissue specific changes to neutrophils (1, 13, 33, 35). Olaloye et al. investigated the role of neutrophils in small intestine from infants with NEC (13). A significant finding was the inverse relationship between circulating and intestinal neutrophils in S-NEC cases, revealing that neutrophils present in the intestinal mucosa had recently migrated from the bloodstream (11). Patients with higher neutrophil levels in the intestine exhibited lower circulating neutrophil counts which is consistent with studies in peripheral blood highlighted above (1, 33, 35). Notably, the active recruitment of neutrophils by the chemoattractant chemokine ligand 8 (CXCL8 also known as IL-8) to inflamed intestinal tissues, contributing to localized inflammation (13). Similarly, Liu et al. reported an increased abundance of neutrophils in intestinal tissues of infants with NEC that release cytokines, including IL-1a, IL-1β, and TNF-α, which amplified the inflammatory response and likely contribute to disease progression by exacerbating local tissue damage and recruiting additional immune cells to the site of inflammation (12). These studies confirm the presence and pro-inflammatory role of neutrophils within NEC-affected intestinal tissues, linking their local activation to both systemic neutropenia and disease progression.

Mechanistic insight into neutrophil involvement in NEC has been provided by Heuer et al. using a novel human intestinal organoid–neutrophil coculture model (38). This study demonstrated that intestinal epithelial cells derived from NEC patients exhibit heightened TLR4 expression and increased apoptosis following LPS stimulation (38). The presence of neutrophils further potentiated epithelial injury and TLR4 signaling in LPS-stressed NEC tissue, supporting a model in which neutrophils exacerbate and sustain intestinal inflammation once the NEC inflammatory cascade has been initiated (38). These findings suggest that neutrophils act as pathological amplifiers of intestinal injury in TLR4-primed epithelium, exerting more severe inflammatory effects in intestines with elevated TLR4 expression, as observed in NEC patients (38).

Meanwhile, the impact of manipulating neutrophils on intestinal inflammation has been explored in animal models of NEC. In neonatal mice, NEC was induced by administering granulocyte colony-stimulating factor (G-CSF) to activate and to increase neutrophils mimicking human neonatal NEC (4). It also resulted in increased occurrence of NEC, intestinal tissue damage, and inflammation but did not affect survival (4). Intestinal injury and NEC severity was reduced in mice lacking neutrophil elastase (NE) highlighting the pathogenic role of neutrophil activation (4). Similarly, G-CSF-treated mice had high levels of neutrophil extracellular traps (NETs) (4). NETs are web-like DNA structures expelled from neutrophils via phagocytosis, studded with antimicrobial proteins and histones, capable of immobilizing and eliminating microorganisms (25). While NETs are important for immune defense against pathogens, excessive formation can lead to hyperinflammation and tissue damage, contributing to diseases like NEC and sepsis (3). Degrading neutrophil extracellular traps (NETs) with agents like DNase1 has been shown to significantly reduce gut inflammation, apoptosis of intestinal cells, and intestinal damage (3). In summary, these studies highlight the role of neutrophils in propagating intestinal epithelial damage in human and experimental NEC.

## 3 Phenotypic differences between classical and cardiac NEC

Thus far, this review has focused on “classical” inflammatory NEC which is primarily a disease of prematurity, intestinal dysbiosis, and inflammation, recent studies have distinguished cardiac NEC as a disease with its own clinical and molecular pathogenesis (5, 39–41). Infants with cardiac NEC, which arises in the setting of medical or surgical treatment for cardiac disease, are typically chronologically younger at the time of diagnosis but of greater gestational age, with higher birth weights compared to infants with classical NEC (5). Cardiac NEC is primarily driven by vascular (ischemia/reperfusion) injury rather than the dysbiosis-induced inflammation and tissue injury seen in classical NEC (39) as such neutrophils, monocytes and macrophages may play a different role and have a distinct phenotype in these patients. However, studies exploring the role of these cell types in infants with congenital heart disease who develop NEC remain limited.

Emerging data suggests neutrophil activation may be a defining feature of cardiac NEC. Compared with infants with classical inflammatory NEC, those with cardiac NEC demonstrate higher circulating leukocyte and neutrophil counts (5). One proposed mechanism is increased formation of NETs leading to increased neutrophil activation and eventual hyperinflammation (5, 40). A study by O'Connor et al. validated fecal calprotectin as a noninvasive biomarker of intestinal inflammation in neonates with congenital heart disease, demonstrating significantly higher levels in infants with definitive NEC compared with those with suspected disease (41). Similarly, MacQueen et al. reported elevated fecal calprotectin concentrations at the onset of bowel symptoms in neonates with NEC and further showed that calprotectin is actively exported from neutrophils through NET formation (40). Together, these findings support a neutrophil-driven inflammatory signature in cardiac NEC, in contrast to classical NEC, which is more commonly associated with sepsis-related neutropenia (40, 41). The distinct clinical and molecular profiles of classical and cardiac NEC indicate that they are separate classes driven by unique inflammatory and ischemic pathways, respectively. Considering these differences is necessary to develop diagnostic tools and therapeutic strategies to improve outcomes for neonates with each disease type.

## 4 Other hematologic abnormality in infants with classical NEC

Other hematologic abnormalities are associated with NEC severity and progression. We briefly describe some here but acknowledge that including the extensive body of work on anemia-transfusion associated NEC is beyond the scope of this review (42–44). Thrombocytopenia particularly in infants <28 weeks' gestation strongly associated with S-NEC and increased mortality (33, 45–47). Similarly, severe anemia (hematocrit <25%) at presentation was linked to a higher risk of mortality across all gestations (43, 45, 47). Likewise, infants born at gestational ages between 28 and 32 weeks are more likely to present with pancytopenia following NEC onset (47). Hematological markers based on gestational age are critical for assessing NEC severity, mortality risk, and the need for surgical intervention though universally accepted values are currently lacking.

## 5 Therapeutic interventions targeting myeloid cells in NEC

To reduce the incidence and severity of NEC, future investigations should focus on incorporating therapeutics that target neutrophils, monocytes/macrophages, and MDSCs. Ideally, these therapies should be safe, effective, easily administered, widely available, and have a known mechanism of action. Several approaches have been explored to date, including modulation of macrophage phenotype, administration of bioactive milk-derived factors, probiotics, and anti-inflammatory or anti-cytokine therapies.

Macrophages play a central role in NEC pathogenesis and exist along a functional spectrum, broadly categorized as classically activated pro-inflammatory M1 macrophages and alternatively activated anti-inflammatory M2 macrophages (48). M1 macrophages activate TLR4 signaling and produce pro-inflammatory cytokines that contribute to intestinal injury, whereas M2 macrophages promote immune regulation, epithelial repair, and resolution of inflammation (11, 48). Dysregulation of this balance, with predominance of M1 polarization, has been implicated in NEC development (48). Additionally, inhibition of TLR4 signaling has been shown to alleviate NEC-associated inflammation, thrombocytopenia, and intestinal damage (11). Consequently, therapeutic strategies aimed at suppressing M1 polarization and/or promoting M2 polarization represent a promising avenue for intervention.

Several experimental therapies have been developed to directly target macrophage activity. Semapimod, a macrophage deactivator, suppresses pro-inflammatory cytokine production (49). In neonatal rat models of NEC, administration of semapimod reduced macrophage activation, limited intestinal injury, and partially protected against intestinal epithelial cell death through suppression of pro-inflammatory cytokine signaling both *in vitro* and *in vivo* (49).

Beyond macrophage depletion or deactivation, therapies that actively promote M2 polarization have also shown benefit. Heparin-binding EGF-like growth factor (HB-EGF) suppresses M1 polarization while promoting M2 transition, thereby enhancing mucosal healing (11). Hydrogen therapy has similarly been shown to inhibit NF-κB activation and favor M2 polarization (11). Moreover, glutaredoxin-1 deficiency and deletion of interferon regulatory factor 5 (IRF5) in myeloid cells reduce M1 polarization and systemic inflammation in experimental NEC (11). Collectively, these findings support targeted modulation of macrophage phenotype as a viable therapeutic strategy.

Anti-cytokine therapy offers a promising approach for treating NEC by targeting key inflammatory pathways. Blocking TNF-α signaling with antibodies or inhibitors reduces intestinal inflammation and tissue damage, while tocilizumab, an IL-6 receptor inhibitor, alleviates NEC by blocking IL-6 signaling (11). TNF-α and TGF-β, released by activated intestinal macrophages, play critical roles in NEC pathogenesis. Supplementing TGF-β2 encourages the differentiation of immature, pro-inflammatory macrophages into mature, non-inflammatory ones, reducing NEC incidence (11). In parallel, supplementation with IL-10 and TGF-β enhances FOXP3 expression, suggesting that monocyte dysfunction in NEC may impair regulatory T-cell development and contribute to disease progression (21). Together, these findings elucidate the role of monocytes and macrophages in intestinal inflammation in S-NEC and highlight the therapeutic potential of IL-10 and TGF-β–based strategies (13, 21). Overall, these strategies highlight the therapeutic potential of modulating cytokine activity to prevent intestinal injury and inflammation in NEC.

Similarly, IL-37 modulates immune homeostasis, TLR repertoires, and microbial diversity (50). Both IL-37 and its receptor IL-1R8 are reduced in human NEC epithelia, with lower IL-37 levels in blood monocytes of infants with NEC (50). Transgenic IL-37 mice were effectively protected from NEC-related complications, whereas exogenous IL-37 showed only moderate efficacy in preventing NEC (50). The study suggests that targeting TLRs and IL-37 could offer new therapeutic options for NEC. IL-37 counteracts inflammation, particularly in the small intestine, and restores intestinal TGF-β1 levels. While boosting the IL-37 pathway shows promise, further optimization is needed for effective exogenous IL-37 treatment (50).

Recent experimental studies support therapeutic strategies aimed at restoring MDSC recruitment and function. Inhibition of GIT2, along with modulation of other upstream regulators such as TFF3 and adenosine signaling, enhances intestinal MDSC accumulation resulting in reduced intestinal inflammation, improved barrier integrity, and restoration of adaptive immune balance (10, 31, 32). In murine models, these approaches consistently increase regulatory T-cell responses, suppress Th17-driven inflammation, and improve survival, underscoring the translational potential of targeting MDSC-mediated immune regulation to mitigate NEC severity (10, 31, 32).

Among biologic therapies, recombinant TGF-β2 has attracted interest. TGF-β2 is readily synthesized in large quantities, is naturally present at high concentrations in amniotic fluid and human milk, and is a known suppressor of macrophage cytokine production in NEC affected intestine (51). However, further studies are required to clarify its role as a prophylactic or therapeutic intervention in infants at risk for or affected by NEC.

Maximizing human milk feeding remains one of the most effective strategies for NEC prevention (11). Bioactive components of human milk, including lactoferrin and human milk oligosaccharides, enhance intestinal macrophage immune function and significantly reduce NEC incidence (11). Human milk oligosaccharides promote M2 macrophage activation, leading to reduced intestinal injury in preterm infants (11). Lactoferrin, in particular, plays a critical role in inducing a suppressive phenotype in MDSCs, which regulate inflammatory responses during early microbial gut colonization. This activity counteracts Th17 cell upregulation in the small intestine during NEC while increasing regulatory T cells in animal models (28).

A key limitation of lactoferrin as a therapeutic agent is that it is administered enterally, and infants receiving standard treatment for NEC are often not receiving enteral nutrition (52). Although the use of donor human milk in conjunction with maternal milk is increasing among extremely premature infants at risk for NEC, lactoferrin concentrations vary in maternal milk and are reduced by pasteurization of donor human milk (52). As such, establishing a therapeutic threshold for lactoferrin in human milk and providing targeted supplementation to infants with inadequate exposure may represent a useful direction for future studies.

Adding to this protective evidence, Vakhal et al. demonstrated that human milk derived extracellular vesicles (EVs) carry bioactive micro-RNA molecules that may shield the newborn intestines from inflammation (53). The study found that term-derived EVs were shown to inhibit secretion of IL-6 and reduced the expression of IL-1β in macrophages, two primary drivers of the hyperinflammatory response. Furthermore, both term and preterm milk derived EVs were shown to reduce pyroptosis which is often triggered by the cytokine storm seen in NEC (53).

Probiotics have been extensively researched as a preventative strategy for infants at elevated risk of developing NEC (11, 54, 55). Sajankila et al. highlight how probiotics may help protect against NEC by restoring balance to the gut microbiota and modulating the neonatal immune response (54). Specific strains, such as *Lactobacillus*, have been shown to promote anti-inflammatory M2 macrophage differentiation, potentially reducing intestinal injury associated with NEC (55). Moreover, byproducts of probiotic activity, like butyric acid, contribute to inflammation control by encouraging macrophage maturation and immune regulation (11). Evidence suggests that multi-strain probiotic combinations, particularly those containing both *Lactobacillus* and *Bifidobacterium*, are more effective than single-strain formulations in decreasing NEC incidence and related mortality (54). The protective effects of probiotics may be further amplified when given alongside human milk, which supports more effective gut colonization (54). In addition to probiotics, other strategies such as prebiotics (e.g., human milk oligosaccharides), synbiotics (combinations of probiotics and prebiotics), and postbiotics (bioactive microbial metabolites) are under investigation for their potential to prevent NEC. However, despite encouraging data, routine probiotic use remains controversial especially in the U.S. Concerns persist regarding inconsistent clinical outcomes, the risk of probiotic-associated sepsis, and the absence of formulations approved by the Food and Drug Administration (FDA). While the NEC Society (56) advocates for their cautious use in very low birth weight infants, the American Academy of Pediatrics (57) calls for more robust evidence to determine standardized, safe, and effective probiotic use in this population.

Understanding the interplay between intestinal macrophages and other cells within the intestine including the enteric nervous system (ENS), and intestinal epithelial cells (IECs) is crucial for innovative NEC treatments. Disruptions in intestinal motility, regulated by the ENS and interstitial cells of Cajal (ICCs), increase NEC risk in preterm infants (11). Muscularis macrophages (MMs) and an immature ENS contribute to dysregulated motility, which might be restored by enhancing gut microbiota (9). Additionally, intestinal macrophages influence IECs to produce IL-10 via TLR4 signaling, maintaining epithelial integrity. While TNF-α supports mucosal repair, sustained nitric oxide release during NEC hinders IEC migrate and repair (11). Exploring these interactions could improve NEC therapies while preserving intestinal barrier function.

Early broad spectrum antibiotic exposure can result in intestinal dysbiosis which is known to increase the incidence of NEC (58). On the contrary, Nguyen et al. highlight the therapeutic potential of oral antibiotic administration during the early postnatal days in preterm neonates (59). In this study, preterm pigs received oral broad-spectrum antibiotics, ampicillin (30 mg/kg three times daily), gentamicin (2.5 mg/kg twice daily), and metronidazole (10 mg/kg three times daily), for five days after birth. Oral administration was superior to systemic antibiotic delivery in preventing NEC, as it more effectively enhanced blood neutrophil maturation, reduced gut colonization and permeability, and prevented bacterial translocation, thereby protecting formula-fed preterm infants from bacteremia and NEC-like lesions. While concerns about increased microbial resistance and adverse long-term effects may limit the widespread use of neonatal short-term antibiotics, particularly for preterm newborns, it is crucial to explore novel preventive and therapeutic approaches for these vulnerable pediatric patients. Antimicrobial proteins and peptides in human milk may replicate the effects of oral antibiotics, and there is a need to comprehend the combined effects of early milk and microbiota on the developing gut and immune systems to support the health of preterm infants in both the short and long term (59).

## 6 Discussion

**Figure 2:**
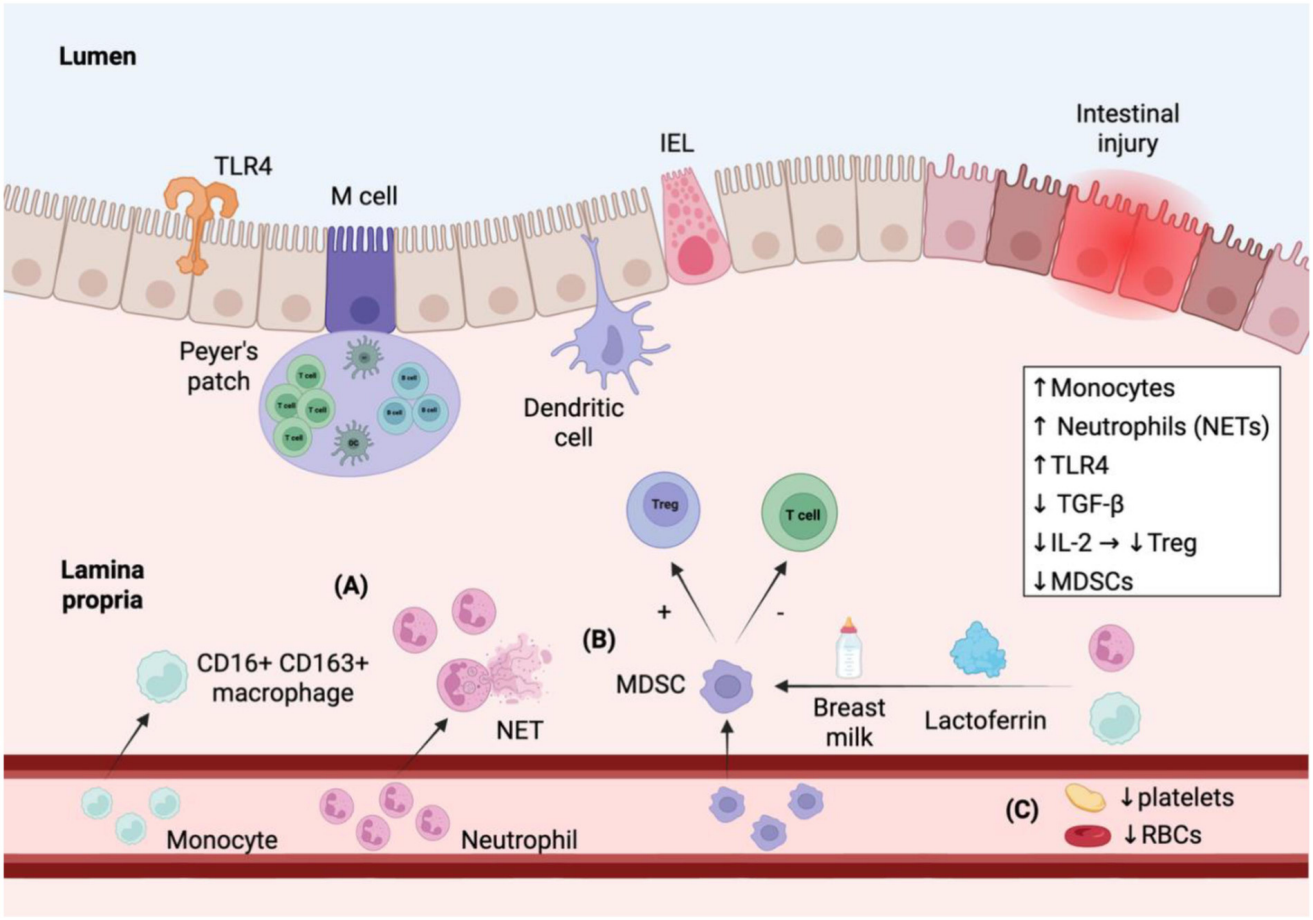
Myeloid trafficking and immune/hematologic dysregulation in classical necrotizing enterocolitis. Schematic overview of the immune, hematologic, and epithelial changes that define NEC pathogenesis and severity. **(A)** At NEC onset, monocytes and neutrophils traffic from the peripheral blood to the intestinal tissue where they differentiate into pro-inflammatory CD16+ CD163+ macrophages and activated neutrophils, respectively. CD16+ CD163+ macrophages are linked to Treg dysfunction and enhanced TLR4 activity in the intestinal tissue. Activated neutrophils result in excessive NET formation leading to epithelial damage and apoptosis. Furthermore, an increase in neutrophil-to-lymphocyte ratio is correlated with NEC severity and risk. Myeloid trafficking results in a decline in absolute monocyte count (AMC) and absolute neutrophil count (ANC) in the peripheral blood which can be used as a reliable clinical marker of disease and severity. **(B)** Regulatory immune pathways are disrupted in NEC. Myeloid-derived suppressor cells (MDSCs) work by inhibiting T cells and activating Treg cells to produce anti-inflammatory cytokines like IL-10 and TGF-β. MDSC levels are reduced in very low birth weight infants and are associated with high NEC risk. Breast milk and derivatives like lactoferrin promote the conversion of neutrophils and monocytes to MDSCs resulting in dampening of inflammatory T cell responses. **(C)** Hematologic abnormalities act as another marker of NEC severity. A decrease in platelets and red blood cells is linked to high mortality risk and may indicate worsening intestinal necrosis. Overall, NEC is linked to general cytopenias reflecting systemic inflammation. IEL—intestinal epithelial cell. Created in BioRender.

Myeloid cells play a pivotal role in the pathogenesis of NEC by driving inflammatory processes that contribute to intestinal injury. Excessive activation of neutrophils and monocytes coupled with impaired MDSC suppressive function is prevalent in NEC affected mucosa. Clinical biomarkers can be obtained from a CBC, as the AMC, NLR, and ANC within 1 week of NEC onset are associated with disease severity. Profound declines in peripheral monocyte and neutrophil counts are correlated with an increase in intestinal neutrophil and monocyte counts in patients with S-NEC. Monocytes and neutrophils are implicated in inflammation-driven intestinal damage and support the exploration of strategies to modulate their activation and inflammatory responses for therapeutic benefit (Supplementary Table S1, Figures 2A,B).

Several therapeutic interventions have shown potential in mitigating monocyte and neutrophil-driven inflammation in NEC. Blocking TLR4 signaling or modulating cytokine responses, such as with TNF-α or IL-6 inhibitors, has been shown to reduce intestinal injury in experimental models. Similarly, human milk EVs can inhibit inflammatory cytokines IL-6 and IL1β. Human milk derived TGF-β2 and lactoferrin promote anti-inflammatory macrophage differentiation and enhance the generation of immunosuppressive myeloid-derived suppressor cells (MDSCs) respectively (Figure 2C). In animal models, lactoferrin-induced MDSCs were more effective than lactoferrin alone, suggesting cell-based therapies may hold future promise. Multi-strain probiotics when administered with breast milk, offer additional protection against NEC by modulating immune responses, suppressing TLR4 signaling, and enhancing Treg activity. These diverse therapeutic strategies highlight a multifaceted approach to NEC prevention and treatment that combines immune regulation, microbial support, and nutritional interventions.

Despite these promising developments, several limitations remain in current research. Many studies are retrospective or rely only on animal models, which may not fully reflect the complexity of NEC in human neonates. Additionally, gaps in understanding the precise molecular interactions between immune cells, the intestinal epithelium, and the microbiota limit the development of targeted therapies. Future research should prioritize prospective, multi-center studies in infants with NEC, employing advanced immunologic and microbiome profiling techniques to uncover precise cellular pathways. Ultimately, a better understanding of the immune mechanisms underlying NEC will guide the development of safe, effective therapies that reduce disease burden while preserving critical immune functions in preterm infants.

## Glossary

AAP: American academy of pediatrics
ALC: absolute lymphocyte count
AMC: absolute monocyte count
ANC: absolute neutrophil count
BM: breast milk
BM-MDSC: breast milk–derived myeloid-derived suppressor cell
CBC: complete blood count
CHD: congenital heart disease
CRP: C-reactive protein
CSF1: colony stimulating factor 1
DNase1: deoxyribonuclease I
ENS: enteric nervous system
EVs: extracellular vesicles
FOXP3: forkhead box P3 (treg transcription factor)
GA: gestational age
GIT2: G-protein–coupled receptor kinase–interacting protein 2
HB-EGF: heparin-binding EGF-like growth factor
ICCs: interstitial cells of cajal
IECs: intestinal epithelial cells
IL: interleukin
IRF5: interferon regulatory factor 5
LF: lactoferrin
LF-MDSC: lactoferrin-induced myeloid-derived suppressor cell
M-NEC: medical necrotizing enterocolitis
MDSC: myeloid-derived suppressor cell
MMs: muscularis macrophages
M-MDSC: monocytic myeloid-derived suppressor cell
NE: neutrophil elastase
NE-SFL: neutrophil fluorescence intensity
NEC: necrotizing enterocolitis
NETosis: process of neutrophil extracellular trap formation
NETs: neutrophil extracellular traps
NF-*κ*B: nuclear factor Kappa-light-chain-enhancer of activated B cells
NLR: neutrophil-to-lymphocyte ratio
NLRP3: NOD-, LRR- and pyrin domain-containing protein 3
NO: nitric oxide
NOS2: nitric oxide synthase 2
NRP1: neuropilin-1
nRP-NEC: non-rapidly progressive necrotizing enterocolitis
PGE2: prostaglandin E2
PMN-MDSC: polymorphonuclear (granulocytic) myeloid-derived suppressor cell
PCT: procalcitonin
PLR: platelet-to-lymphocyte ratio
RP-NEC: rapidly progressive necrotizing enterocolitis
S-NEC: surgical necrotizing enterocolitis
TFF3: trefoil factor 3
TGF-*β*: transforming growth factor beta
Th: T helper cell
Th17: T helper 17 cell
TLR: toll-like receptor
TLR4: toll-like receptor 4
TNF-α: tumor necrosis factor alpha
Treg: regulatory T cell
VLBW: very low birth weight.
