# Supplementary material for: The role of myeloid cells in the pathogenesis of necrotizing enterocolitis; a scoping review
Source: Front Pediatr. 2026 Feb 27;14:1750294. doi: 10.3389/fped.2026.1750294 (PMC12982386; doi:10.3389/fped.2026.1750294)
Supplement: Supplementary file 1 [file Supplementaryfile1.docx]

**Table S1.** Peripheral and Intestinal Immune Alterations in NEC

| **Cell Type / Marker** | **Key Findings in NEC** |
| --- | --- |
| **Monocytes / AMC**^14,15,16,17,18,^ | • ↓ AMC at NEC onset; more profound in S-NEC  • >50–75% reduction predicts Bell Stage III, need for surgery, and progression  • Reflects recruitment to intestine and macrophage differentiation |
| **Intestinal Monocytes / Macrophages (CD16⁺CD163⁺)**^13,21,25^ | • Enriched in NEC lesions; promote pro-inflammatory environment  • Trafficking from blood → explains AMC decline  • Linked to Treg dysfunction and enhanced TLR4 activity |
| **Transforming Growth Factor-β (TGF-β)**^20,,51^ | • ↓ circulating TGF-β1 levels predict NEC risk (<1,380 pg/mL) • ↓ intestinal TGF-β2 bioactivity worsens mucosal injury  • Recombinant TGF-β2 is protective in neonatal NEC models |
| **Interleukin-2 (IL-2)**^20^ | • Reduced IL-2 impairs Treg survival and homeostasis → ↑ intestinal inflammation |
| **MDSCs (PMN-MDSCs & M-MDSCs)**^28,29,30^ | • Lower in VLBW infants and associated with higher NEC risk • Breast milk ↑ MDSC suppressive function → ↓ inflammation, ↓ bacterial load, ↑ survival in models  • Reduced S100A8-A9 signaling → ↓ monocyte stress tolerance |
| **Lactoferrin-Induced MDSCs**^2,28^ | • LF in breast milk converts neutrophils/monocytes → MDSCs  • Powerful dampening of T-cell responses and tissue inflammation  • Candidate preventive/therapeutic pathway in NEC |
| **Neutrophils / ANC**^1,33^ | • ↓ ANC predicts rapid progression and mortality  • Included in severity prediction models (AUC ~0.80)  • Reflects massive trafficking to intestine/peritoneum |
| **Neutrophil-to-Lymphocyte Ratio (NLR)**^35^ | • ↑ NLR correlates with NEC severity and risk  • Proposed predictive window: 1.60–3.20 |
| **Intestinal Neutrophils**^1,8^ | • Elevated in NEC tissue  • Inverse blood:tissue neutrophil levels support active recruitment |
| **Neutrophil Extracellular Traps (NETs)**^3,4^ | • Excessive NET formation → epithelial damage and apoptosis  • DNase1 degradation of NETs reduces disease severity in models |
| **Platelets (Thrombocytopenia)**^45,46,47^ | • Strongly associated with S-NEC and mortality, especially in <28w GA  • Often progressive → may indicate worsening intestinal necrosis and surgical need |
| **Red blood cells / Anemia**^42,43,44,46,47^ | • Severe anemia (hematocrit <25%) linked to higher mortality at all gestational ages  • Anemia-transfusion interactions are important but beyond review scope |
| **Pancytopenia Pattern (GA-Dependent)**^47^ | • Infants 28–32w GA more likely to show multi-lineage cytopenias after NEC onset  • Indicates systemic inflammatory involvement and potentially severe disease |
| **Gestational-Age-Dependent Lab Norms**^14,46,47^ | • No universal cut-offs → interpretation must consider GA-specific baselines for risk stratification |
| **Fecal Calprotectin**^40,41^ | • Significantly elevated at symptom onset in definitive NEC vs. suspected NEC  • Distinguish NEC from feeding intolerance and other gastrointestinal issues |

**Table S2.** Summary of Key Human Immunologic Studies in NEC

| **Author / Year** | **Study Type** | **Focus** | **Key Findings** |
| --- | --- | --- | --- |
| **Mu & Wang, 2022**^35^ | Retrospective case-control | Neutrophil-to-Lymphocyte Ratio (NLR) | Higher NLR values in infants with NEC; useful inflammatory biomarker with strong sensitivity and specificity |
| **Wang, Chong et al., 2023**^14^ | Retrospective case-control | Absolute Monocyte Count (AMC) | Sharp AMC decline at NEC onset correlates with severity; lower in S-NEC vs. M-NEC; supports role of AMC in predicting surgical need |
| **Tajalli et al., 2022**^9^ | Case-control | Monocyte levels | ↓ circulating monocytes with ↑ tissue monocyte infiltration in NEC; supports monocytes as early diagnostic biomarker |
| **Gordon et al., 2016**^46^ | Cohort | CBC abnormalities by GA | Severe anemia and thrombocytopenia associated with mortality; acute monocyte drop at NEC onset; reinforced GA-specific interpretation |
| **Chen et al., 2023**^1^ | Retrospective cohort | Rapid progression model | Model incorporating male sex, portal venous gas, low ANC, and pH predicted rapid progression; ↓ ANC reflects neutrophil migration to bowel |
| **Garg et al., 2022**^47^ | Retrospective cohort | GA-specific hematologic patterns | <28w: high WBC/ANC/AMC + thrombocytopenia  28–32w: more pancytopenia Thrombocytopenia <32w linked to mortality |
| **Olaloye et al., 2023**^13^ | Case-control | CD16⁺CD163⁺ monocytes | Elevated pathogenic monocyte subset in blood and bowel; ↑ TLR4/IL-6/IL-8; biomarker and potential therapeutic target in S-NEC |
| **Pantalone et al., 2021**^8^ | Case-control | CBC by GA and NEC type | <33w: monocyte/lymphocyte fall + band rise in S-NEC  >33w: acute neutrophil decline  Monocyte rebound during recovery |
| **Y. Liu et al., 2023**^12^ | Case-control | Immune signatures (CyTOF) | Enrichment of non-classical monocytes, neutrophils, and CD4⁺ memory cells in NEC mucosa; MDSCs reduced - protective role |
| **Pang et al., 2018**^21^ | Case-control | Monocyte-T cell cytokine axis | NEC monocytes show ↑ TLR4, TNF-α, IL-6 and ↓ IL-10, TGF-β → drives Th17>Treg imbalance and inflammation |
| **Chong et al., 2024**^29^ | Retrospective cohort | Hematologic predictors of severity | <28w S-NEC: lower platelets  <32w: drops in lymphocytes, monocytes, platelets accurately distinguish S-NEC |
| **Maheshwari et al., 2014**^20^ | Case-control | Cytokine biomarkers | TGF-β1 <1,380 pg/mL predicted NEC risk (64% accuracy); first biomarker for NEC susceptibility |
| **Desiraju et al., 2020**^18^ | Retrospective cohort | AMC & severity | Greater AMC decline in Stage III NEC; supports AMC as disease-severity biomarker |
| **Köstlin et al., 2018**^30^ | Case-control | Breast milk MDSCs | Breast milk-derived MDSCs downregulate TLR4 and help prevent NEC |
| **Qin et al., 2022**^33^ | Retrospective cohort | Neutropenia & mortality | ↓ ANC strongly associated with S-NEC and death; ΔANC most sensitive predictor of surgical NEC (71.7%) |
| **Liu et al., 2025** ^36^ | Retrospective cohort | Neutrophils, leukocytes, pH | Acidic pH strongly associated with mortality in both surgical and non-surgical NEC  Higher neutrophil and WBC count independently associated with lower pH  Neutrophil count negatively correlated with pH |
| **Guo et al., 2024** ^34^ | Retrospective cohort | ANC as severity predictor | Significantly higher ANC within 24 h of onset in surgical/death NEC vs medical NEC  ANC identified as an independent predictor of surgical or fatal NEC  Composite predictive model (ANC + PLR + CRP + PCT) showed good discrimination (AUC 0.79) |
| **Ferraro et al., 2024** ^37^ | Retrospective cohort | Neutrophil activation and maturation (NE-SFL) | NE-SFL significantly elevated at sepsis/NEC onset  NE-SFL outperformed ANC, band count, I/T ratio, and CRP  Identifies NE-SFL as a sensitive, real-time biomarker for NEC-associated inflammation |
| **Heuer et al., 2025** ^38^ | Case-control | Neutrophil–epithelial interaction, TLR4 signaling | NEC-derived epithelium shows increased TLR4 expression and apoptosis after LPS  Neutrophils exacerbate epithelial injury and NEC-like changes in TLR4-high organoids |
| **Klinke et al., 2021** ^5^ | Retrospective cohort study | Classical NEC vs. cardiac NEC | Cardiac NEC infants are older with high birthweights and show higher neutrophil counts compared to classical NEC |
| **O’Connor et al., 2020** ^41^ | Prospective cohort study | Fecal calprotectin in congenital heart disease | ↑ fecal calprotectin in neonates with congenital heart defects (>3500 µg/g) in patients who developed NEC compared to those with suspected NEC |
| **MacQueen et al., 2016** ^40^ | Prospective cohort study | Calprotectin & NETs | ↑ fecal calprotectin in neonates with cardiac NEC is linked to activated neutrophils releasing NETs |
| **Burge et al., 2025** ^39^ | In vitro study | Classical NEC vs. cardiac NEC | Classical NEC shows dysbiosis induced inflammation and metabolic acidosis  Cardiac NEC shows ER stress induced apoptosis and reduced angiogenesis |
| **Bisht et al., 2025** ^17^ | Retrospective cohort study | AMC in NEC vs feeding intolerance | ↓ AMC in NEC (p=0.074)  ↑ AMC in infants with feeding intolerance (p < 0.001) |
| **Moroze et al., 2024** ^16^ | Retrospective cohort study | AMC & diagnosis | Rapid↓ AMC precedes clinical signs of NEC. Studying velocity of ↓ AMC provides earlier diagnostic window than static AMC levels |
| **Vakhal et al., 2025** ^53^ | In vitro study | Human milk extracellular vesicles (EVs) | Milk EVs deliver microRNA that inhibit IL-6 and IL-1β (upregulated in NEC) secretion in macrophages thereby protecting the intestinal epithelium from injury; provides a molecular mechanism for human milk’s protective effect in NEC |
| **Sun et al., 2024** ^22^ | In vitro study | Epithelial-Myeloid Signaling | Epithelial-derived CSF1 is essential for maintaining macrophage homeostasis; downregulation in NEC leads to ↑ inflammatory CSF1R+ fetal macrophages that drive tissue injury via the TLR4/NF-κB pathway |

**Table S3.** Summary of Key Immunology Animal Studies in NEC

| **Author / Year** | **Study Type** | **Focus** | **Key Findings** |
| --- | --- | --- | --- |
| **Klinke, Vincent et al., 2020**^4^ | Case-control (murine model) | Neutrophil activation & NETs | • G-CSF–induced neutrophil activation increased NEC incidence and severity  • Greater intestinal injury accompanied by excess NET formation • Neutrophil elastase expression markedly elevated in NEC tissue  • Mice lacking neutrophil elastase were almost completely protected from NEC → demonstrating neutrophils as key drivers of pathology |
| **Nguyen, Fuglsang et al., 2016**^59^ | Case-control (preterm pig model) | Neutrophil maturation & microbiome | • Oral antibiotics promoted neutrophil maturation, reduced bacteremia, and lowered NEC rates  • Effect likely related to delayed gut bacterial colonization and improved early immune readiness • Suggests that immature neutrophil responses contribute to NEC susceptibility in preterm neonates |

**Table S4.** Summary of Key Immunology Combined Studies in NEC

| **Author / Year** | **Study Type** | **Focus** | **Key Findings** |
| --- | --- | --- | --- |
| **He et al., 2018**^28^ | Case-control | MDSCs & lactoferrin | • Neonatal MDSCs are critical for controlling early intestinal inflammation  • Lactoferrin (LF) induces MDSC suppressive activity through nitric oxide, PGE2, and S100A8-A9  • Higher MDSC activity in normal-birth-weight infants vs. VLBW infants  • LF expands MDSCs → ↓ Th17 / ↑ Tregs → reduced intestinal inflammation  • MDSC-based therapies show potential for NEC prevention/treatment |
| **Maheshwari et al., 2011**^51^ | Case-control | TGF-β in intestinal immunity | • NEC is marked by reduced TGF-β expression in the intestine  • Loss of TGF-β → worsened mucosal injury and inflammation  • Recombinant TGF-β2 protects against NEC-like injury in models  • TGF-β in human milk may contribute to NEC protection (vs. formula)  • Supports evaluating enteral TGF-β2 as a prophylactic intervention |
| **Liu et al., 2019**^2^ | Case-control | LF-induced MDSC therapy | • Low early-life MDSCs predict NEC development  • LF converts neutrophils/monocytes → PMN-MDSCs & M-MDSCs  • LF-MDSCs > LF alone in reducing inflammation, bacterial burden, and mortality  • Highlights therapeutic promise of LF-MDSC transfer |
| **Cho et al., 2020**^50^ | Case-control | IL-37 immunoregulation | • Transgenic IL-37 reduces NEC intestinal injury in mice  • Supports IL-37 as a novel therapeutic target for NEC |
| **Le et al., 2024** ^10^ | Case-control | GIT2 regulation of MDSC recruitment | GIT2 expression increased in human and experimental NEC and correlated with injury severity  Git2 deletion protected against NEC via enhanced intestinal MDSC recruitment  Identifies GIT2 inhibition as a potential therapeutic strategy |
| **Liu et al., 2021**^31^ | Case-control | TFF3-mediated PMN-MDSC activation | TFF3 activated and expanded PMN-MDSCs via NF-κB/COX2/PGE₂ signaling  TFF3 or TFF3-induced PMN-MDSCs reduced inflammation, permeability, and bacterial translocation |
| **Zhou et al.,**  **2022** ^32^ | Case-control | Adenosine in MDSC function | Adenosine treatment significantly attenuated NEC severity and mortality  Enhanced MDSC immunosuppressive and antibacterial activity  Identifies adenosine–MDSC axis as a therapeutic target |
| **Balamurugan et al., 2025** ^23^ | Experimental study | Integrin β3 & Platelets | Platelet-monocyte aggregation via integrin β3 drives injury; knockout reduces inflammatory cytokine levels and severity of intestinal injury |
| **Zheng et al., 2024** ^26^ | Experimental study | Regulatory monocytes (NRP1+) | Identified unique NRP1- high monocyte subset that protects against neonatal inflammation |
